# Course and prediction of body image dissatisfaction during pregnancy: a prospective study

**DOI:** 10.1186/s12884-022-05050-x

**Published:** 2022-09-20

**Authors:** Katja Linde, Franziska Lehnig, Michaela Nagl, Holger Stepan, Anette Kersting

**Affiliations:** 1 Department of Psychosomatic Medicine and Psychotherapy, University of Leipzig, Semmelweisstraße 10, 04103 Leipzig, Germany; 2 Leipzig University Medical Center, IFB AdiposityDiseases, Philipp-Rosenthal-Straße 27, 04103 Leipzig, Germany; 3 Department of Obstetrics, University of Leipzig, Liebigstraße 20a, 04103 Leipzig, Germany

**Keywords:** Body image, Pregnancy, Depression, Self-esteem, Worry, Sleep quality, BMI

## Abstract

**Background:**

During pregnancy, women`s bodies undergo rapid changes in body weight and body size within a relatively short period of time. Pregnancy may therefore be associated with an increased vulnerability for the development of body image dissatisfaction that has been linked to adverse health outcomes for mother and child. The present study aims to examine changes in body image during pregnancy as well as predictors of body image dissatisfaction. This is the first study using a tailored, multidimensional measure of body image especially developed for the pregnant population.

**Methods:**

A prospective longitudinal design with a quantitative approach was applied. Healthy pregnant women (*N* = 222) were assessed using standardized instruments at two time points (T1: 18th-22th week of gestation, T2: 33th-37th week of gestation). The impact of demographic, weight- and health-related, behavioral, and psychological factors assessed at T1 on body image dissatisfaction at T1 and T2 was examined using stepwise linear regression analyses.

**Results:**

T-tests for paired samples revealed that dissatisfaction with strength-related aspects of body image, dissatisfaction with body parts, and concerns about sexual attractiveness increased significantly from the middle to the end of pregnancy. In contrast, preoccupation with appearance, dissatisfaction with complexion, and prioritization of appearance over function were significantly reduced over time. Stepwise linear regression analyses revealed that factors influencing body image depend on the component of body image investigated. Overall, a low level of self-esteem and a high level of pregnancy-specific worries were risk factors for several components of body image dissatisfaction. Besides these, poor sleep quality, low levels of physical activity, disturbed eating behavior, and higher levels of BMI and weight gain were significant predictors.

**Conclusions:**

The results highlight the multidimensional nature of body image and show positive as well as negative changes during pregnancy. Overall, modifiable psychological, behavioral, and weight-related factors appear relevant to the extent of body image dissatisfaction.

**Supplementary Information:**

The online version contains supplementary material available at 10.1186/s12884-022-05050-x.

## Background

During pregnancy, women`s bodies undergo rapid changes in body weight and body size within a relatively short (40-week) period of time. These unique physical changes are likely to contribute to a reevaluation of women`s body image [1] and may promote body image dissatisfaction [2]. Therefore, pregnancy occurs to be an ideal time to investigate body image changes as well as factors leading to body image dissatisfaction.

The term body image refers to an individual’s internal representation of his or her outer appearance [3]. There is consensus that body image is a multidimensional construct compromising cognitive, emotional, perceptual, and behavioral components [4]. The term body image dissatisfaction is part of an attitudinal component of body image relating to the degree of dissatisfaction associated with specific aspects of the body [5]. Body image dissatisfaction during pregnancy has often been associated with adverse maternal and child health outcomes [6, 7]. For instance, body image dissatisfaction was linked to depressive symptoms, unfavorable dieting behavior and eating disorders, excessive gestational weight gain, and postpartum weight retention [6, 7]. It is furthermore proposed to influence parent–child interactions, including problematic child feeding practices, which may in turn be associated with child self-regulation, child eating behavior, and risk of childhood obesity [6].

The number of studies investigating the course of body image dissatisfaction from pre-pregnancy to pregnancy or during pregnancy prospectively are rare and findings are inconsistent. Some studies suggest an increase in body image dissatisfaction from the time before pregnancy to the first or last trimester of pregnancy for the whole sample [8–10] or a subgroup of pregnant women without eating disorders [11]. In contrast, one study suggests a decrease in body image dissatisfaction from the time before pregnancy to the last trimester of pregnancy for a subgroup of high exercising women [12]. Furthermore, some studies suggest stability of body image dissatisfaction during pregnancy for the whole sample [8, 13], or at least for a subgroup of low exercising pregnant women [12] and pregnant women with eating disorders [11]. Besides differences in study design (e.g., number of measurement points before and during pregnancy), reporting changes within subgroups [11, 12], and comparisons done (time before pregnancy to pregnancy or different measurement points within pregnancy), these inconsistencies may also reflect individual differences in internalization of body image ideals. Associations between internalization of the thin ideal and body image dissatisfaction were shown in the general population [14] and the postpartum period [15, 16]. Another important reason for inconsistencies between the studies might be the use of different measures of body image dissatisfaction, making comparisons across studies difficult. While body image is described as a multifaceted construct, most research has focused solely on the degree of satisfaction with appearance which was measured along a single continuum [8–11, 13]. So far, only two studies applied a multidimensional measure to investigate the course of body image dissatisfaction. The results indicate stability, as well as an increase and decrease of body image dissatisfaction during pregnancy depending on the component of body image dissatisfaction, examined [2, 17]. For instance, women felt less strong during pregnancy compared to before pregnancy [2, 17] but stronger at the end compared to mid-pregnancy [17]. They felt significantly less fat at the end of pregnancy compared to pre-pregnancy, the beginning, and the middle of pregnancy [2, 17]. Concerning attractiveness and salience of weight and shape, results were inconsistent. One study showed no change in the salience of weight and shape but a significant decline in attractiveness from pre-pregnancy to the middle of pregnancy [2] whereas another study showed a decline in the salience of weight and shape but no significant changes in attractiveness [17].

Besides multidimensionality, body image measures that were used within the population of pregnant women were mostly developed for and validated among non-pregnant populations. They often did not cover body parts (e.g., skin, breast, stomach) or features (functionality, sexual attractiveness) that become more relevant in pregnant compared to non-pregnant populations [18, 19]. To the best of our knowledge, there is no study so far using a multidimensional and specific measure of body image dissatisfaction during pregnancy.

Results from studies in the general population suggest that correlates contributing to body image dissatisfaction seem to be multi-factorial and bio-psycho-social [3, 6, 20]. However, factors influencing body image dissatisfaction during pregnancy have not been systematically investigated yet [1]. One review [1] considering demographic, physical, sociocultural, psychological, and behavioral correlates within the population of pregnant women concluded that psychological factors play an important role. Hereby, the surfeit of studies focused on depression, indicating a robust but weak positive association with body image dissatisfaction in cross-sectional as well as longitudinal designs and for different measures [2, 17, 21, 22]. Another review concluded that depression influences only some parts of body image during pregnancy [7]. Other psychological correlates like stress [21], eating restraints [23, 24] and commitment to pregnancy [21] showed weak positive associations with body image dissatisfaction while social support seems to be a protective factor [21]. In addition, a lifetime history of mental disorders [25] and self-esteem [26–28] were related to body image during pregnancy in more recent studies not included in the review mentioned above. Due to the small, often single number of cross-sectional studies, the relationships require replication. Studies investigating the impact of socio-demographic factors such as age, income, partnership, or school education are scarce and samples are often homogenously making comparisons difficult [29]. One cross-sectional study [30] reported an effect of parity, indicating that primiparous women regarded their appearance in late pregnancy as more positive than multiparous women. The association between physical (sleep quality, physical symptoms, weight gain, BMI) and behavioral factors (exercise, smoking) with body image dissatisfaction seem to be small and inconsistent [29]. To sum up, except for depression, the number of studies investigating correlates of body image dissatisfaction during pregnancy is small, and results either need replication or seem to be inconsistent. The majority of studies used cross-sectional designs that limit the potential to draw causal interferences and may contribute to an overestimation of effect sizes [29]. Multivariate longitudinal studies using a multidimensional and specific measure of body image dissatisfaction are needed to extend the knowledge about risk factors of body image dissatisfaction during pregnancy and to develop targeted interventions.

## Aims

The first aim was to investigate changes in body image dissatisfaction during pregnancy (i.e., from the second to the third trimester of pregnancy) using a prospective study design and a tailored, multidimensional measure of body image especially developed for the pregnant population. The measure covers key features of body image in pregnancy, including body dissatisfaction, importance and ideals of body image, pregnancy-related body changes, functioning of the pregnant body, sexual attractiveness, and appearance-related behaviors [31]. The second aim was to systematically investigate the influence of sociodemographic factors, pregnancy-, weight- and physical health-related factors as well as behavioral, social, and psychological factors on body image dissatisfaction using a multivariate cross-sectional as well as prospective design. The influence of distal and proximal correlates on the different dimensions of body image dissatisfaction in middle (cross-sectional, T1) and late pregnancy (prospective, T2) was examined. Due to the small number of previous studies as well as weak or inconsistent findings an exploratory procedure was used to examine the best set of influencing factors.

## Methods

### Participants and procedure

The study was conducted according to the Declaration of Helsinki and was approved by the Ethics Committee of the Medical Faculty of the University of Leipzig (reference number: 422/17-ek, 14.11.2017). Participants were recruited at the Department of Obstetrics of the University of Leipzig (Germany) while waiting for routine prenatal diagnostic appointments. Recruitment took place between April 2018 and December 2019. Eligible women were pregnant, above 18 years of age, between the 18^th^ and 22^th^ week of gestation, and provided written informed consent. Women with multiple pregnancies and inadequate German reading or writing skills to answer the questionnaires were excluded from the study. Women who agreed to take part in the study were given study information and an informed consent sheet. According to their choice participating women were handed out the first study questionnaire and a prepaid return envelope, or they received an email link to the online version of the first questionnaire at the time of recruitment. Data for the present analyses were collected at two assessment points during pregnancy as part of a larger prospective study: T1 (second trimester: 18^th^-22^th^ week of gestation) and T2 (third trimester: 33^th^-37^th^ week of gestation). At each assessment point, women were invited to fill out the paper–pencil or online version of the study questionnaire. Non-responders were contacted by email or postal mail up to two times within a three-week time frame.

### Measures

Body image: The German version of the Body Image in Pregnancy scale (BIPS [31, 32]) was used to measure multiple components of body image in pregnancy. The German version of the BIPS consists of 32 items to be answered on a 5-point response scale. It covers six dimensions of body image: preoccupation with appearance, dissatisfaction with strength-related aspects, dissatisfaction with body parts, dissatisfaction with complexion, prioritization of appearance over function, and concerns about sexual attractiveness. Higher scores indicate higher body disturbances. The German version is a reliable and valid measure [32]. The internal consistency of the subscales in the present sample was as follows: preoccupation with appearance (T1: α = 0.91, T2: α = 0.90), dissatisfaction with strength-related aspects (T1: α = 0.89, T2: α = 0.90), dissatisfaction with body parts (T1: α = 0.82, T2: α = 0.84), dissatisfaction with complexion (T1: α = 0.83, T2: α = 0.88), prioritization of appearance over function (T1: α 0.87, T2: α = 0.83), and concerns about sexual attractiveness (T1: α = 0.80, T2: α = 0.79).

Weight- and health-related factors: Pre-pregnancy body weight (in kg) was assessed via retrospective self-report at T1. Certainty of retrospective assessment was assessed with a single item using a 10-point response scale [median = 9, modus = 10, range 1 ‘not safe at all’ to 10 ‘extremely safe’]. Actual body weight (in kg) was assessed via self-report at T1 and T2. Body height (in m) was assessed via self-report at T1. Weight gain was calculated by subtracting body weight at T1 from body weight at T2. Pre-pregnancy body mass index was calculated by dividing pre-pregnancy body weight (in kg) by the square of body height (in m). The occurrence of current physical disorders was measured with a dichotomous self-generated item [‘Do you currently suffer from any kind of physical disorders?’].

Pregnancy-related factors: The intensity of planning and desiring the current pregnancy was measured with two self-generated items [‘To which degree did you plan the current pregnancy?’, ‘How intense was your desire to be pregnant?’) on a 5-point response scale. Higher values indicate a higher intensity of planning and desiring for the current pregnancy.

Behavioral factors: Frequency of light, moderate, and intense physical activity during the past seven days prior to the assessment was measured with three single items of the German version of the short form of the International Physical Activity Questionnaire (IPAQ [33]). Higher values indicate a higher frequency of light, moderate and intense physical activity. The number of days with eating attacks and the number of days with uncontrolled eating were measured using two single items of the German version of the Eating Disorders Examination-Questionnaire (EDE-Q [34, 35]). The EDE-Q covers eating disorder psychopathology during the last 28 days prior to the assessment. The scores of both items range from 0 to 1, with the value 1 indicating the presence of eating attacks and uncontrolled eating, respectively.

Psychological factors: History of mental disorders before pregnancy was assessed through a dichotomous self-generated question [‘Have you ever been diagnosed with a mental disorder before you became pregnant?’]. Current depressive symptoms were assessed using the German version of the Edinburgh Postnatal Depression Scale (EPDS [36, 37]). The EPDS sum score ranges from 0 to 30 with higher scores indicating a higher severity of depressive symptomatology. A cutoff of $\ge$ 10 has been established during the second trimester of pregnancy to indicate significantly elevated levels of depression [38]. The German version of the EPDS has been shown to have a good reliability [37] and the applicability of the EPDS for the use during pregnancy has been established [38]. In the current sample, the internal consistency of the EPDS sum score was α = 0.84 at T1. Self-esteem was measured with the revised German version of the 10-item Rosenberg Self-Esteem Scale (RSE [39]). The RSE sum score ranges from 0 to 30 with higher scores indicating higher self-esteem. The reliability and validity of the German version of the RSE were acceptable [40]. The internal consistency of the RSE sum score in the present sample was α = 0.88 at T1. Content and extent of worries in pregnancy were measured using the German version of the 17-item Cambridge Worry Scale (CWS [41, 42]). The CWS sum score ranges from 0 to 85 with higher scores indicating a greater extent of pregnancy-related worries. There is evidence for the reliability and validity of the German version in pregnant women [41]. The internal consistency of the CWS sum score in the present sample was α = 0.85 at T1. Sleep quality was measured with the German version of the Pittsburgh Sleep Quality Index (PSQI [43, 44]. The PSQI total score ranges from 0 to 21 with higher scores indicating more reduced sleep quality. A cutoff of > 5 has been established to indicate poor sleep quality [43]. There is evidence for the reliability and validity of the PSQI total score [43, 45, 46]. The internal consistency of the PSQI total score in the present sample was α = 0.66 at T1. Received social support was measured with the Berlin Social Support Scales (BSSS [47]). The mean score of the subscale Received Social Support ranges from 0 to 4 with higher scores indicating a greater extent of received social support. There is evidence for the reliability and validity of the BSSS [47]. The internal consistency of the subscale Received Social Support in the present sample was α = 0.87 at T1.

Sociodemographic variables were measured using self-generated questions. Variables included maternal age, partnership, education, household income, and parity.

### Statistical analyses

Data analyses were performed using the Statistical Package for Social Sciences, version 24.0.0.2 (IBM® SPSS®), and the significance level was set to 0.05. Before main analyses missing values and drop-out analyses were performed. Drop-out analyses were performed using independent t- (continuous variables) or X^2^-tests (categorical variables).

T-tests for paired samples were conducted to investigate the transition of body image over time (T1 to T2). Bonferroni–Holm method [48] was used to counteract the problem of multiple testing of the six indicators of body image. Wilcoxon-test was conducted to investigate the change in frequency of women scoring above the cutoff of the BSQ at T1 and T2 assessments.

Bivariate correlations between the outcome measures (body dissatisfaction, preoccupation with appearance, dissatisfaction with strength-related aspects of the pregnant body, dissatisfaction with body parts, dissatisfaction with complexion, prioritization of appearance over function, concern about sexual attractiveness) and the other study variables at T1 and T2 were calculated using Pearson coefficient (for continuous variables) or point-biserial coefficient (for binary variables). Variables that showed a significant correlation with any of the outcome measures were entered as independent variables in a stepwise linear regression analysis separately for the six outcome measures at T1 and T2. Variables were included if they were significant at *p* < 0.05 and excluded if *p* was > 0.10. The model with the highest explained variance (R^2^ and adjusted R^2^ respectively) was selected (final model). For the diagnosis of multicollinearity, the variance inflation factor was calculated, considering values above 5 as an indication of multicollinearity. The assumption of independent errors was checked using the Durbin-Watson statistic. The assumptions of normal distribution was visually checked by using a histogram of errors. The assumptions of heteroscedasticity was checked by using the White test [49].

## Results

**Fig. 1:**
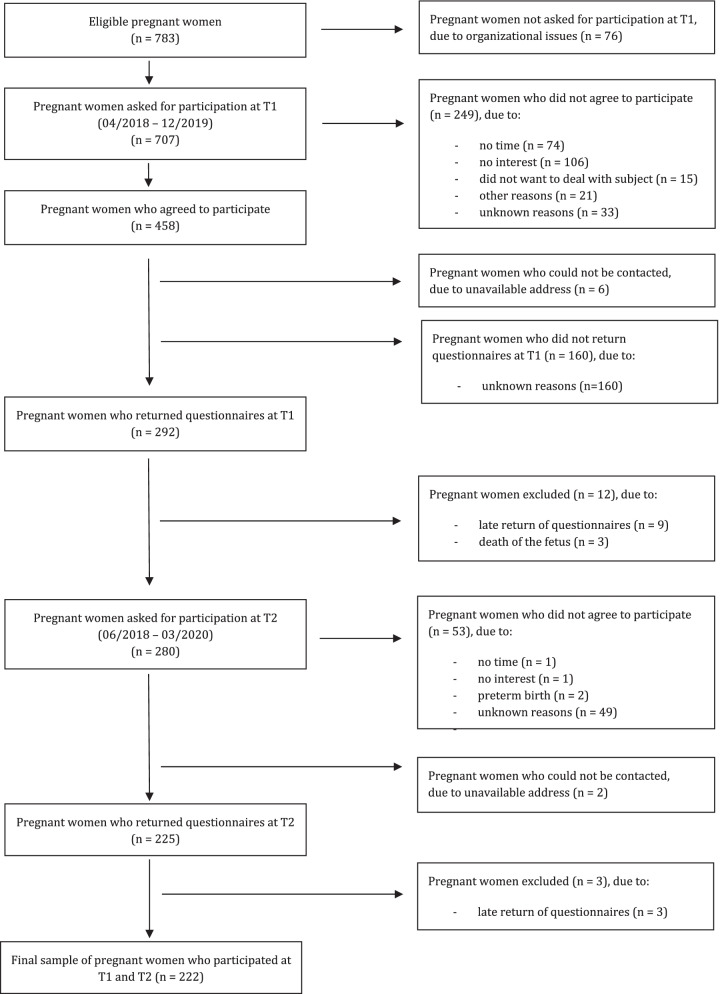
Flow diagram

A total of 783 women were eligible for study entry. Of them, 452 agreed to participate (57.7%) and 292 (37.3%) returned T1 questionnaires (see Fig. 1). For the current analyses, 12 women were excluded at T1 due to late return of questionnaires or death of the fetus, resulting in *N* = 280 women participating at T1. Of them, 225 returned T2 questionnaires. For the current analyses, 3 women were excluded at T2 due to the late return of questionnaires resulting in a final sample of *N* = 222 women participating at T1 and T2. The number of drop-outs from T1 to T2 was 58 (26.1%). Drop-out analyses revealed significant differences for education [χ^2^(2) = 20.060, *p* = 0.000] and income [t(274) = -2.576, *p* = 0.011] only. Women who dropped out of the study were less educated and had a lower household income.

Table 1 shows the sample characteristics at T1 assessment. Participants’ age ranged from 19 to 45 years. The majority of women were of German nationality, had a high level of school education and were in a partnership. Nearly half of the women had given birth to one or more children before. More than half of the sample had a household income of less than 3.000€. 17.1% of women reported suffering from any mental disorders in their lifetime, most often from depression or anxiety disorders. 23.0% of women reported elevated depression symptoms and 49.8% poor sleep quality during the second trimester (T1). Nearly half of the women reported suffering from physical disorders during the second trimester (T1), most often from thyroid disease, followed by bowel disease and hypertension.

**Table 1:** Sample characteristics at T1 assessment

Sociodemographic characteristics	
Age, *M* (*SD*)	31.44 (4.82)
Nationality: German, *n* (%)	214 (96.4)
Education	
low secondary qualification, *n* (%)	6 (2.7)
high secondary qualification, *n* (%)	66 (29.7)
university entrance qualification, *n* (%)	150 (67.6)
Partnership, *n* (%)	218 (98.2)
Household Income	
≤ 1000, *n* (%)	13 (5.9)
1001–2000, *n* (%)	55 (24.8)
2001–3000, *n* (%)	46 (20.7)
3001–4000, *n* (%)	54 (24.3)
4001–5000, *n* (%)	38 (17.1)
≥ 5001, *n* (%)	16 (7.2)
Parity	
Primiparous, n (%)	114 (51.4)
Multiparous, n (%)	108 (48.6)
Weight and physical health	
Current physical disorders, n (%)	101 (45.5)
Pre-pregnancy BMI [kg/m^2^], *M (SD)*	24.64 (5.29)
Weight gain (Pre-pregnancy to T1) [kg], *M (SD)*	4.97 (3.46)
Physical activity and eating behavior	
Number of days with light physical activity, *M (SD)*	5.61 (1.78)
Number of days with moderate physical activity, *M (SD)*	2.57 (2.36)
Number of days with intense physical activity, *M (SD)*	1.38 (1.91)
Eating attacks, n (%)	86 (38.7)
Uncontrolled eating, n (%)	34 (15.3)
Maternal health	
History of mental disorders, n (%)	38 (17.1)
Depression: EPDS $\ge 10$, n (%)	51 (23.0)
Poor sleep quality: PSQI > 5, n (%)	109 (49.8)

*Total sample N* 220, *M* mean, *SD* Standard deviation

### Transition of body image dissatisfaction during pregnancy

Table 2 presents the means and standard deviation of the six pregnancy-specific indicators of body image at T1 and T2 and the results of the two-tailed dependent t-tests without und with correction for alpha level inflation. Dissatisfaction with strength-related aspects of body image, dissatisfaction with body parts, and concerns about sexual attractiveness increased significantly, while preoccupation with appearance, dissatisfaction with complexion, and prioritization of appearance over function was significantly reduced over time. Effect sizes were small, with the largest reduction in prioritization of appearance over function and dissatisfaction with complexion. After correction for alpha-inflation, the results of the significance tests did not change.

**Table 2:** Descriptive statistics of body image variables and test statistics of dependent t-tests

	T1	T2				
	*M* (*SD*)	*M* (*SD*)	*t* (*df* = 221)	*p*	*p*^a^	*d*
**Pregnancy specific measure of body image (BIPS)**						
Preoccupation with appearance	2.12 (0.98)	2.00 (0.90)	2.47	.007	**.014**	-0.13
Dissatisfaction with strength-related aspects	2.72 (0.95)	3.03 (0.95)	-4.99	.000	**.000**	0.33
Dissatisfaction with complexion	2.32 (1.03)	1.95 (1.00)	6.01	.000	**.000**	-0.36
Dissatisfaction with body parts	1.91 (0.83)	2.18 (0.97)	-5.11	.000	**.000**	0.30
Prioritization of appearance over function	2.86 (0.92)	2.52 (0.82)	5.62	.000	**.000**	-0.39
Concerns about sexual attractiveness	2.31 (0.93)	2.40 (0.93)	-1.85	.033	**.033**	0.10

*M* Mean, *SE* Standard error, *SD* Standard deviation, *d* Cohen`s d

Higher values indicate higher levels of body image dissatisfaction

^a^Bonferroni-Holm-correction for multiple testing

### Predictors of body image dissatisfaction during pregnancy

Two additional tables (see Additional files 1 and 2) provide an overview of correlations between the six outcome measures at T1 and T2 and each of the independent variables assessed at T1. With exception of partnership and light physical activity, all other variables significantly correlated with at least one of the six outcome variables at T1 or T2 and were therefore included as independent variables in stepwise linear regression analyses. There was no indication of multicollinearity, autocorrelation, non-normality, or heteroscedasticity of residuals.

Results of the stepwise linear regression analyses for each of the six outcome measures at T1 assessment are presented in Table 3 (cross-sectional analyses). Overall, the final models explained between 2.4% and 34.5% of the variance in indicators of body image at T1. According to Cohen [50], the explanation of variance corresponded to a large-sized effect for preoccupation with appearance and concerns about sexual attractiveness, a moderate-sized effect for dissatisfaction with body parts and dissatisfaction with strength-related aspects, and a small-sized effect for dissatisfaction with complexion and prioritization of appearance over function.

**Table 3:** Summary of the final model of stepwise linear regression analyses of body image at T1

	**BIPS T1**					
	Preoccupation with appearance	Dissatisfaction with strength-related aspects	Dissatisfaction with complexion	Dissatisfaction with body parts	Prioritization of appearance over function	Concerns about sexual attractiveness
	*β*	*β*	*β*	*β*	*β*	*β*
Sociodemographic variables						
Age			-.220**			
School education (0 = low to middle; 1 = high)						
Household income						
Parity (0 = primiparous; 1 = multiparous)				.129*		
Pregnancy-related variables						
Pregnancy plans						
Desire for Pregnancy					-.154*	-.189**
Weight- and physical health-related variables						
Pre-pregnancy BMI				.168**		
Weight gain (before pregnancy to T1)	.148**	.149*				
Current physical disorders (0 = no; 1 = yes)			.163*			.117*
Poor Sleep Quality (PSQI)	.144*			.134*		
Eating- and activity-related variables						
Number of days with eating attacks (EDE-Q)				.201**		
Number of days with uncontrolled eating (EDE-Q)	.331***					.133*
Moderate physical activity (IPAQ)	.122*					
Intense physical activity (IPAQ)	.139*	-.144*				
Psychological variables						
Social Support (BSSS)		-.153*				
Mental disorders before pregnancy (0 = no; 1 = yes)	.140*					
Depression (EDPS)		.222**				
Worry (CWS)			.164*	.205**		
Self-Esteem (RSE)	-.237***					-.426***
*F* (*df1*, *df2*)	16.784*** (7, 214)	8.573*** (4, 217)	7.948*** (3, 218)	8.267*** (5, 216)	5.317* (1, 220)	20.183 (4, 217)
***R***^**2**^** (adjusted *R***^**2**^**)**	**.345 (.333)**	**.136 (.121)**	**.099 (.086)**	**.161 (.141)**	**.024 (.019)**	**.271 (.258)**

Independent and dependent variables were assessed at T1 (cross-sectional analyses). The final model of stepwise linear regression analyses is shown. Higher BSSS scores indicate higher levels of social support. Higher EPDS scores indicate more severe depressive symptomatology. Higher CWS scores indicates higher levels of pregnancy-specific worries. Higher RSE scores indicate higher levels of self-esteem

*β* Standardized regression coefficient

^*^p < .05; **p < .01; ***p < .001

Significant predictors of higher levels of preoccupation with an appearance at T1 were higher weight gain, lower quality of sleep, a higher number of days with uncontrolled eating, more days of moderate as well as intense physical activity per week, suffering from mental disorders before pregnancy and lower level of self-esteem. Significant predictors of higher levels of dissatisfaction with strength-related aspects at T1 were higher weight gain, fewer days of intense physical activity per week, lower levels of social support, and higher levels of depression. Significant predictors of higher levels of dissatisfaction with complexion at T1 were younger age, suffering from one or more current physical disorders, and a higher level of pregnancy-specific worries. Significant predictors of higher levels of dissatisfaction with body parts at T1 were having one or more children, a higher pre-pregnancy BMI, lower quality of sleep, a higher number of days with eating attacks, and a higher level of pregnancy-specific worries. The only significant predictor of higher levels of prioritization of appearance over function at T1 was a lower level of desire for the current pregnancy. Significant predictors of higher levels of concerns about sexual attractiveness at T1 were a lower level of desire for the current pregnancy, suffering from one or more current physical disorders, more days with uncontrolled eating, and a lower level of self-esteem.

Results of the stepwise linear regression analyses for each of the six outcome measures at T2 assessment are presented in Table 4 (prospective analyses). Overall, the final models explained between 8.1% and 33.1% of the variance in indicators of body image at T2. According to Cohen [50], the explanation of variance corresponded to a large-sized effect for preoccupation with appearance, a moderate-sized effect for concerns about sexual attractiveness and dissatisfaction with strength-related aspects, and a small-sized effect for dissatisfaction with body parts, dissatisfaction with complexion and prioritization of appearance over function.

**Table 4:** Summary of the final model of stepwise linear regression analyses of body image at T2

	**BIPS T2**					
	Preoccupation with appearance	Dissatisfaction with strength-related aspects	Dissatisfaction with complexion	Dissatisfaction with body parts	Prioritization of appearance over function	Concerns about sexual attractiveness
	*β*	*β*	*β*	*β*	*β*	*β*
Sociodemographic variables						
Age						
School education (0 = low to middle; 1 = high)						
Household income			-.139*			
Parity (0 = primiparous; 1 = multiparous)						
Pregnancy-related variables						
Pregnancy plans						
Desire for Pregnancy						
Weight-and physical health-related variables						
Pre-pregnancy BMI				.180**		
Weight gain (before pregnancy to T1)	.119*			.147*		
Current physical disorders (0 = no; 1 = yes)	.341***					
Poor Sleep Quality (PSQI)	.133*	.241***				.147*
Eating- and activity-related variables						
Number of days with eating attacks (EDE-Q)				.135*		
Number of days with uncontrolled eating (EDE-Q)						.216***
Moderate physical activity (IPAQ)	.160**				-.160*	
Intense physical activity (IPAQ)		-.158*				
Psychological variables						
Social Support (BSSS)						-.141*
Mental disorders before pregnancy (0 = no; 1 = yes)		.178**				
Depression (EDPS)						
Worry (CWS)	.232***		.249***	.211**	-.200**	
Self-Esteem (RSE)	-.161*				-.268***	-.311***
*F* (*df1*, *df2*)	17.700*** (6,215)	11.216*** (3,218)	10.538*** (2,219)	7.966*** (4, 217)	6.396*** (3, 218)	16.516*** (4, 217)
***R***^**2**^** (adjusted *R***^**2**^**)**	**.331 (.312)**	**.134 (122)**	**.088 (.079)**	**.128 (.112)**	**.081 (.068)**	**.233 (.219)**

Independent variables were assessed at T1. Dependent variables were assessed at T2 (prospective analyses). The final model of stepwise linear regression analyses is shown. Higher EPDS scores indicate more severe depressive symptomatology. Higher CWS scores indicates higher levels of pregnancy-specific worries. Higher RSE scores indicate higher levels of self-esteem

*β* Standardized regression coefficient

^*^p < .05; **p < .01; ***p < .001

Significant predictors of higher levels of preoccupation with appearance at T2 were higher weight gain from pre-pregnancy to T1, suffering from one or more current physical disorders, lower quality of sleep, more days with moderate physical activity per week, higher level of pregnancy-specific worries and a lower level of self-esteem at T1. Significant predictors of higher levels of dissatisfaction with strength-related aspects at T2 were lower quality of sleep, fewer days with intense physical activity per week, and suffering from one or more mental disorders. Significant predictors of higher levels of dissatisfaction with complexion at T2 were lower household income and higher levels of pregnancy-specific worries at T1. Significant predictors of higher levels of dissatisfaction with body parts at T2 were a higher pre-pregnancy BMI, higher weight gain from pre-pregnancy to T1, higher number of days with eating attacks, and higher levels of pregnancy-specific worries at T1. Significant predictors of higher levels of prioritization of appearance over function at T2 were fewer days with moderate physical activity per week, lower levels of pregnancy-specific worries, and lower levels of self-esteem. Significant predictors of higher levels of concerns about sexual attractiveness at T2 were lower quality of sleep, more days with uncontrolled eating, lower levels of social support as well as self-esteem at T1.

## Discussion

The first aim of the study was to investigate changes in body image dissatisfaction during pregnancy, using a tailored, pregnancy-specific, and multidimensional measure of body image. Overall, significant but small-sized changes in all six components of body image dissatisfaction were found. Results showed a decrease in preoccupation with appearance, dissatisfaction with complexion, and prioritization of appearance over function from mid-pregnancy to the end of pregnancy, but an increase in dissatisfaction with strength, dissatisfaction with body parts, and concerns about sexual attractiveness. The course of body image dissatisfaction during pregnancy seems to depend on the component investigated. On the one hand, women adapted to changes in their bodies as they were more satisfied with their appearance, functionality, and complexion at the end of pregnancy compared to mid-pregnancy. So far, only two other studies [2, 17] support an improvement of appearance-related aspects of body image (“feeling fat”) over the course of pregnancy. On the other hand, women in the present study were less satisfied with their fitness (e.g. strength, flexibility, muscle tone) and specific body parts that underlie changes as pregnancy progresses (e.g. hands, ankles, chest). Furthermore, they suffered more from feelings of not being sexually attractive and avoided showing themselves naked to others at the end compared to mid-pregnancy. A decline in strength and fitness during pregnancy is supported by one study [2], whereas another study found an increase [17]. Concerning sexual attractiveness, two studies showed no significant changes during pregnancy, but they used a global measure of attractiveness instead of focusing especially on sexuality [2, 17]. Overall, comparisons between studies are difficult to make because this is the first study using a pregnancy-specific, multidimensional measure of body image during pregnancy. In contrast, the majority of previous studies used unidimensional measures of body image [8–13], compared body image during pregnancy with pre-pregnancy body image only [9–12], or used an unspecific but multidimensional measure [2, 17] with components of body image that differs from the questionnaire used in this study.

The second aim of our study was to simultaneously investigate sociodemographic, pregnancy-, weight- and physical health-related as well as psychological correlates of body image in pregnancy using cross-sectional and prospective analyses.

First of all, the chosen predictors explained relevant amounts of variance in four out of six components of body image dissatisfaction (preoccupation with appearance, concerns about sexual attractiveness, dissatisfaction with strength, and dissatisfaction with body parts) in cross-sectional as well as prospective analyses. The chosen predictors seemed to be less relevant for dissatisfaction with complexion and prioritization of appearance over function. Physiological or hormonal factors [51] could be more predictive of satisfaction with facial complexion, hair or skin tone, and the functionality of the body, breasts, or stomach than the ones applied in the current study.

Second, there was no single predictor or set of predictors that was important for each of the six components of body image uniformly, indicating that predictors vary depending on the dimension of body image investigated. This result was also found in another study and supports the multidimensional conceptualization of body image [2].

Third, socio-demographic variables and pregnancy-related attitudes seemed to be less relevant than weight- and physical health-related variables, eating- and activity-related variables, and psychological variables, which was also supported by a review [1].

Fourth, the importance of predictors varied depending on the time of assessment of body image. Hence, some variables were significant predictors in cross-sectional analyses only, some in prospective analyses, and others were significant predictors of one or more components of body image dissatisfaction in both analyses. The corresponding variables will be discussed in more detail below.

Predictors like age, parity, desire for pregnancy, suffering from one or more mental disorders before pregnancy, depression as well as social support were significant predictors in cross-sectional analyses only, whereas household income and suffering from one or more physical disorders were significant predictors in prospective analyses only. The first mentioned factors may play a role in the evaluation of body image in the middle of pregnancy. Interestingly, these factors lose their impact at the end of pregnancy. It is to be seen whether this trend could be replicated by future studies.

Concerning socio-demographic variables, the results confirm previous research showing that having one or more children [30] is associated with less body image dissatisfaction, at least dissatisfaction with body parts. Furthermore, the results add to previous research by showing that higher age and having a higher household income protect against dissatisfaction with complexion. Older women might place less value on the appearance of skin and hair while being pregnant and women having more money might easier be able to compensate for skin and hair changes using specialized cosmetic products. Concerning commitment to pregnancy, the current results confirm one cross-sectional study [21] showing that a higher desire for pregnancy is associated with less body image dissatisfaction, especially fewer concerns about sexual attractiveness and less prioritization of appearance. Considering physical health-related and psychological variables, cross-sectional analyses confirm previous research [1] by showing that having physical problems, receiving low levels of social support [21], and suffering from mental disorders, especially depression, is associated with higher body image dissatisfaction.

Predictors which were significant in cross-sectional as well as prospective analyses indicate causal and more robust relationships. Concerning psychological variables, to the best of our knowledge, this is the first study showing that global self-esteem and pregnancy-specific worries were predictors of several components of body image dissatisfaction in late pregnancy. Women with lower levels of self-esteem seem to evaluate the appearance and attractiveness-related aspects of body image worse. A negative self-image and a low feeling of self-worth seem to be predictive of a negative body evaluation of pregnant women. This was also supported by other studies [26, 52]. Furthermore, women who experience higher levels of pregnancy-specific worries (e.g., money or health problems, problems with relatives, worries about birth or hospital) seem to be more dissatisfied with complexion and body parts, and more preoccupied with their appearance but tend to worry less about appearance when directly compared to the functionality of their body (e.g., breasts, stomach). These results are in line with a previous cross-sectional study [21], showing that stress and body image were negatively related to pregnancy. However, the results need to be verified by future studies. Concerning eating- and activity-related variables, results show that moderate and intense physical activity, as well as eating behavior, play a role in some specific aspects of body image during pregnancy. Concerning physical activity, results are controversial. Whereas a higher number of days with intense physical activity (e.g., aerobic, running, fast cycling, and swimming) protected against dissatisfaction with strength-related aspects, a higher number of days with moderate physical activity (e.g., carrying of light loads, cycling, and swimming with ordinary speed) was associated with a stronger preoccupation with appearance. Light physical activity (e.g., walking) was not related to body image at all. It seems that only intense physical activity is a relevant protective factor of body image in pregnant women. Results are partly in line with other studies [12, 22] showing that women reporting higher pre-pregnancy exercise levels were more satisfied with their bodies in late pregnancy than low-exercising women. Concerning eating behavior, a higher number of days with eating attacks was a specific risk factor for dissatisfaction with body parts in late pregnancy and a higher number of days with uncontrolled eating was a specific risk factor for concerns about sexual attractiveness. Results extend prior studies by showing that not only restraint eating but also excessive eating might be a risk factor for body image during pregnancy [23, 53]. Concerning weight- and physical health-related variables study results extend findings from a cross-sectional study [26] by showing that poor global sleep quality during mid-pregnancy was a relevant risk factor for several components of body image dissatisfaction in late pregnancy, especially for strength, appearance, and sexual attractiveness. According to Kamysheva et al. [26] the findings might be mediated by reduced psychological well-being caused by high levels of fatigue. In the current study, nearly half of the sample suffered from poor sleep quality, and about one-fifth of women suffered from prior mental disorders and actual depressive symptoms in mid-pregnancy. Concerning weight-related variables, the BMI before pregnancy was a risk factor for dissatisfaction with body parts in late pregnancy. Weight gain from pre-pregnancy to mid-pregnancy was also a risk factor for dissatisfaction with body parts as well as dissatisfaction with appearance in prospective analyses. These results confirm other study results by showing that weight-related factors play a role in body image dissatisfaction [1], even if they seem to be only relevant for some body image components and not for all.

Comparing the relevance of influencing predictors, weight- and physical health-related as well as eating- and activity-related variables seem to be less relevant than psychological factors. Overall, psychological variables showed stronger associations with body image dissatisfaction and were related to more components of body image in pregnancy.

### Strengths and limitations

The major strength of the study was the use of a tailored, pregnancy-specific multidimensional questionnaire for body image in pregnancy. Thereby it was possible to investigate the course of diverse components of body image in detail. In addition, the use of a prospective study design extended previous findings from cross-sectional studies and allowed to make causal interferences concerning potential risk factors for body image dissatisfaction during pregnancy. Furthermore, multivariate analyses of a wide range of different influencing factors contributed to previous findings by showing which predictors are most relevant for body image during pregnancy.

The predominant limiting factor of the present study is the use of only two measurement points during pregnancy. Therefore, the current results only refer to the course of body image from the second to the third trimester. Further studies should investigate the course of body image from the beginning to the end of pregnancy and ideally include pre-pregnancy body image to determine which changes could be attributed uniquely to pregnancy. Besides this, the majority of women in the current sample were in their early thirties, had a stable partnership, and were well educated, which reduces the generalizability of results. The homogeneity of the sample may also have led to a reduced variation in demographic and other variables, resulting in small or non-significant associations with body image. Results should therefore be replicated in a more diverse sample. Furthermore, due to the exploratory nature used for stepwise linear regression analyses, results need to be replicated in studies based on a hypothesis-driven method. Last, physical activity and eating behavior were assessed using single items and self-reports, which might limit the reliability of these constructs. This also applies to the retrospective assessment of height and weight to calculate the pre-pregnancy BMI. Furthermore, the reliability of the total score of the PSQI was within a questionable to a nearly acceptable range. Therefore results need to be interpreted with caution and should be replicated in further studies.

## Conclusions

In conclusion, study findings indicate different courses for different components of body image during pregnancy, although changes were rather small. As pregnancy progress and women experience rapid changes in weight, shape, and physical symptoms, they seem to adapt to some changes but suffer from others. The findings confirm that BMI, weight gain, social support, physical activity, and uncontrolled or excessive eating play a role in some components of body image. Furthermore, the findings highlight the importance of global self-esteem, pregnancy-specific worries, and global sleep quality as risk factors for several components of body image dissatisfaction in pregnant women. Health care professionals should be aware of and ask for these risk factors. Due to the associations of an impaired body image during pregnancy with the development of depressive symptoms, disturbed eating behavior and postpartum weight retention as well as known negative effects on children’s health, interventions should be developed. Based on study results interventions should consist of psychological components aiming to influence self-esteem and worries as well as behavioral components promoting better sleep quality, eating behavior, and physical activity.

## Supplementary Information

**Additional file 1.**

**Additional file 2.**

## Data Availability

The datasets used for analysis during the current study are available from the corresponding author on request.
