# Supplementary material for: Course and prediction of body image dissatisfaction during pregnancy: a prospective study
Source: BMC Pregnancy Childbirth. 2022 Sep 20;22:719. doi: 10.1186/s12884-022-05050-x (PMC9487034; doi:10.1186/s12884-022-05050-x)
Supplement: Supplementary file 1 — Additional file 1. [file 12884_2022_5050_MOESM1_ESM.docx]

**Additional File 1** Bivariate correlations of potential predictors assessed at T1 with body image components at T1

|  |  | **BIPS T1** | | | | | | |
| --- | --- | --- | --- | --- | --- | --- | --- | --- |
|  |  | Preoccupation with appearance | Dissatisfaction with strength-related aspects | Dissatisfaction with complexion | Dissatisfaction with body parts | Prioritization of appearance over function | Concerns about sexual attractiveness |  |
| Sociodemographic variables | |  |  |  |  |  |  |  |
|  | Age | .054 | -.013 | **-.201**** | .047 | .002 | .008 |  |
|  | Partnership (yes vs. no) | -.022 | -.083 | -.001 | -.034 | -.001 | -.069 |  |
|  | School education (low to middle vs. high) | .077 | -.035 | -.101 | -.050 | -.109 | .002 |  |
|  | Household income | -.076 | -.071 | **-.166*** | **-.191**** | -.054 | -.108 |  |
|  | Parity (nulliparous vs. > nulliparous) | .044 | -.090 | **.146*** | -.085 | -.110 | -.051 |  |
| Pregnancy-related variables | |  |  |  |  |  |  |  |
|  | Pregnancy plans | -.077 | -.047 | -.063 | .017 | -.106 | -.122* |  |
|  | Desire for pregnancy | **-.131*** | -.080 | -.002 | -.099 | **-.154*** | **-.214**** |  |
| Weight-and physical health-related variables | |  |  |  |  |  |  |  |
|  | Pre-pregnancy BMI | .077 | .060 | -.067 | .210** | **.129*** | .101 |  |
|  | Weight gain (before pregnancy to T1) | **.228**** | **.172**** | .012 | .118* | -.037 | **.178**** |  |
|  | Current physical disorders (yes vs. no) | **.114*** | .022 | **.157**** | .110 | .082 | **.114*** |  |
|  | Sleep Quality (PSQI) | **.172**** | **.177**** | **.166**** | **.208**** | .036 | **.199**** |  |
| Eating- and activity-related variables | |  |  |  |  |  |  |  |
|  | Number of days with eating attacks (EDE-Q) | **.185**** | .095 | .093 | **.254**** | .015 | **.124*** |  |
|  | Number of days with uncontrolled eating (EDE-Q) | **.342**** | .059 | **.127*** | **.202**** | .013 | **.257**** |  |
|  | Light physical activity (IPAQ) | -.009 | -.004 | -.039 | -.018 | .047 | .031 |  |
|  | Moderate physical activity (IPAQ) | **.213**** | .052 | .002 | .025 | -.030 | **.114*** |  |
|  | Intense physical activity (IPAQ) | **.179**** | **-.158**** | -.069 | -.030 | **.126*** | .074 |  |
| Psychological variables | |  |  |  |  |  |  |  |
|  | Social Support (BSSS) | **-.181**** | **-.193**** | -.104 | -.096 | -.104 | **-.214**** |  |
|  | Mental disorders before pregnancy (yes vs. no) | **.251**** | **.122*** | .034 | .049 | .006 | **.170**** |  |
|  | Depression (EPDS) | **-.181**** | **-.193**** | -.104 | -.096 | -.104 | **-.214**** |  |
|  | Worry (CWS) | **.272**** | **.264**** | **.169**** | **.160**** | .057 | **.251**** |  |
|  | Self-Esteem (RSE) | **-.373**** | **-.209**** | **-.188**** | **-.177**** | -.088 | **-.447**** |  |

Notes. Pearson respectively point-biserial correlation coefficient with significance level, **p*<.05; ***p*<.01
