# Supplementary material for: Course and prediction of body image dissatisfaction during pregnancy: a prospective study
Source: BMC Pregnancy Childbirth. 2022 Sep 20;22:719. doi: 10.1186/s12884-022-05050-x (PMC9487034; doi:10.1186/s12884-022-05050-x)
Supplement: Supplementary file 2 — Additional file 2. [file 12884_2022_5050_MOESM2_ESM.docx]

**Additional File 2** Bivariate correlations of potential predictors assessed at T1 with body image components at T2

|  |  | **BIPS T2** | | | | | | |
| --- | --- | --- | --- | --- | --- | --- | --- | --- |
|  |  | Preoccupation with appearance | Dissatisfaction with strength-related aspects | Dissatisfaction with complexion | Dissatisfaction with body parts | Prioritization of appearance over function | Concerns about sexual attractiveness |  |
| Sociodemographic variables | |  |  |  |  |  |  |  |
|  | Age | **-.123*** | -.014 | **-.127*** | .085 | .011 | **-.116*** |  |
|  | Partnership | -.021 | .017 | .032 | .003 | .072 | -.017 |  |
|  | School education (low to middle vs. high) | .029 | -.082 | -.104 | -.067 | **-.131*** | -.001 |  |
|  | Household income | **-.156*** | -.040 | **-.163**** | **-.157**** | **-.134*** | **-.136*** |  |
|  | Parity (nulliparous vs. > nulliparous) | .104 | -.018 | .109 | -.048 | -.077 | .009 |  |
| Pregnancy-related variables | |  |  |  |  |  |  |  |
|  | Pregnancy plans | **-.180**** | -.038 | .042 | -.015 | -.090 | **-.154*** |  |
|  | Desire for pregnancy | **-.162**** | -.017 | .086 | -.039 | -.038 | **-.168**** |  |
| Weight-and physical health-related variables | |  |  |  |  |  |  |  |
|  | Pre-pregnancy BMI | .054 | .096 | .029 | **.164**** | .003 | .062 |  |
|  | Weight gain (before pregnancy to T1) | .**203**** | .079 | -.027 | **.166**** | -.096 | **.161**** |  |
|  | Current physical disorders | .067 | .107 | .035 | **.121*** | .082 | .077 |  |
|  | Sleep Quality (PSQI) | **.202**** | **.271**** | **.161**** | **.116*** | -.011 | **.200**** |  |
| Eating- and activity-related variables | |  |  |  |  |  |  |  |
|  | Number of days with eating attacks (EDE-Q) | **.316**** | .064 | .089 | **.219**** | .010 | **.158**** |  |
|  | Number of days with uncontrolled eating (EDE-Q) | **.446**** | .068 | **.160**** | **.136*** | .025 | **.306**** |  |
|  | Light physical activity (IPAQ) | -.026 | -.074 | .004 | .010 | .056 | .048 |  |
|  | Moderate physical activity (IPAQ) | **.176**** | -.068 | -.035 | -.026 | **-.126*** | .067 |  |
|  | Intense physical activity (IPAQ) | .069 | **-.180**** | **-.111*** | -.043 | .008 | .054 |  |
| Psychological variables | |  |  |  |  |  |  |  |
|  | Social Support (BSSS) | **-.156**** | -.031 | -.050 | -.078 | .013 | **-.213**** |  |
|  | Mental disorders before pregnancy (yes vs. no) | **.160**** | **.224**** | .004 | **.143*** | .030 | **.181**** |  |
|  | Depression (EPDS) | **.278**** | **.190**** | **.227**** | **.132*** | .089 | **.270**** |  |
|  | Worry (CWS) | **.328**** | **.231**** | **.262**** | **.239**** | -.074 | **.273**** |  |
|  | Self-Esteem (RSE) | **-.348**** | **-.229**** | **-.202**** | **-.116*** | **-.171**** | **-.386**** |  |

*Notes*. Pearson respectively point-biserial correlation coefficient with significance level, **p*<.05; ***p*<.01
